# Detection of Virulence Factors and Antibiotic Resistance among *Klebsiella pneumoniae* Isolates from Iran

**DOI:** 10.1155/2023/3624497

**Published:** 2023-02-14

**Authors:** Sara Davoudabadi, Mehdi Goudarzi, Ali Hashemi

**Affiliations:** Department of Microbiology, School of Medicine, Shahid Beheshti University of Medical Sciences, Tehran, Iran

## Abstract

The current study assessed the detection of virulence genes and drug resistance among *Klebsiella pneumoniae* isolates from Iran. During 2018 to 2020, 52 *K. pneumoniae* isolates were obtained from patients at Iran hospitals. By disk diffusion method, the antimicrobial susceptibility of *K. pneumoniae* isolates was assessed, and ESBL-producing *K. pneumoniae* isolates were detected by CDDT method. PCR analysis was done to detect virulence genes (*iucB*, *iutA*, *iroN*, *kfu*, *allS*, *fimH*, *ybtS*, *mrkD*, and *entB*); ESBL-encoding genes (*bla*_TEM_, *bla*_PER_, *bla*_CTX-M_, *bla*_VEB_, and *bla*_SHV_); and class D (*bla*_OXA-48_), class B (*bla*_VIM_, *bla*_NDM_, and *bla*_IMP_), and class A (*bla*_KPC_ and *bla*_GES_) carbapenemase genes. Among all isolates, 84.6%, 13.5%, and 1.9% isolates were multidrug-resistant (MDR), extensively drug-resistant (XDR), and pandrug-resistant (PDR), respectively. Also, 84.6% were ESBL-producing and 71.2%, 53.8%, 40.4%, and 9.6% of all isolates were carrying *bla*_TEM_, *bla*_SHV_, *bla*_CTX-M_, and *bla*_OXA-48_ genes, respectively. Six isolates (11.5%) were positive for *bla*_NDM_ gene. In contrast, no isolates were positive for the presence of *bla*_KPC_, *bla*_IMP_, and *bla*_VIM_. Virulence factor genes including *iucB*, *iutA*, *iroN*, *kfu*, *allS*, *fimH*, *ybtS*, *mrkD*, and *entB* were carried by 24%, 46.2%, 25%, 11.5%, 17.3%, 86.5%, 75%, 88.5%, and 100% isolates, respectively. This study evaluated the distribution and prevalence of virulence factor genes among *K. pneumoniae* isolates. The treatment of these infections is challenging due to the existence of particular virulence factors and the rise of antibiotic resistance. Therefore, the current study accentuates the necessity of finding new and efficient solutions for stopping the increase of antibiotic resistance.

## 1. Introduction

*K. pneumoniae* is a facultative anaerobic bacillus which is Gram-negative, encapsulated, and ferment lactose [1, 2], and as it may cause serious infections and life-threatening disorders, this opportunistic pathogen remains a serious concern among public health.

*K. pneumoniae* is a common pathogen among nosocomial infections, including bacteremia, urinary tract infections (UTIs), pneumonia, pyogenic liver abscesses, burn, and wound infections [3, 4]. Additionally, the treatment of *K. pneumoniae* infections is demanding due to the emergence of particular virulence factors as well as the rise of antibiotic resistance [1].

*K. pneumoniae*'s pathogenicity is related to the existence of virulence genes that encode specific virulence factors which let the bacterium target the immune system, resulting in a variety of disorders. Among *K. pneumoniae* isolates, quite a few virulence factors, such as iron acquisition systems and adhesions, which contribute to virulence and pathogenicity, have been introduced [5, 6].

Bacteriocin biosynthesis (yersiniabactin (*ybts*) and enterobactin (*entB*)), type 3 and type 1 adhesins (*fimH* and *mrkD*), aerobactin synthase genes (*iuc* and *iut*), iron acquisition system-related genes (*iron* and *kfu*), and allantoin metabolism-associated gene (*allS*) are virulence-associated genes which have a critical impact in invasion and adhesion of *K. pneumoniae* isolates to host tissues, playing an essential part in the pathogenicity of *K. pneumoniae* isolates collected from the infections which are acquired from the hospital [1, 5–7].

Iron acquisition mechanisms are crucial for pathogenic bacteria's growth. Besides, bacteria can absorb protein-bound iron from the infected cells thanks to the siderophores [1, 5–7].

Above all, antibiotic resistance is growing among *K. pneumoniae* isolates. Gram-negative bacteria, like *K. pneumoniae*, have acquired a variety of antibiotic resistance mechanisms, including the gaining of antibiotic resistance genes, to combat routinely used antimicrobials. The rate of resistance to widely used antibiotic groups, including tetracyclines, aminoglycosides, penicillins, lincosamides, macrolides, folic acid inhibitors, phenicols, and fluoroquinolones, according to epidemiological studies, was high among *K. pneumoniae* strains which were isolated from the infections which are sorted as hospital-acquired [5, 6, 8, 9].

Due to the major impact of virulence genes on the pathogenesis of *K. pneumoniae* isolates and concerning increasing rates of antibiotic resistance among them, this study was to detect the distribution of virulence genes and drug resistance among *K. pneumoniae* isolates from Iran.

## 2. Materials and Methods

### 2.1. Ethical Statement

This report is ethically approved and verified with the code “IR.SBMU.MSP.REC.1398.755,” by SBMU Ethics Committee.

The privacy of the patients was preserved by keeping participants confidential, and identifiable private data was neither acquired nor involved in the research.

### 2.2. Bacterial Isolates

Totally, 52 *K. pneumoniae* isolates were obtained from Iran between October 2018 and September 2020. The identification of bacterial isolates was performed by standard microbiological and biochemical techniques, including the ornithine decarboxylase (OD) test, urease test, methyl red/Voges-Proskauer (MR/VP) test, triple sugar iron agar media (TSI), citrate utilization test, and reactions on SH2/motility/indole (SIM) [10]. Then, bacteria isolates were kept and stored in TSB (HiMedia, India) accompanied with 20% glycerol at -70°C until they were needed.

### 2.3. Antimicrobial Susceptibility Testing

According to the Clinical and Laboratory Standards Institute's standards (CLSI 2020), antimicrobial susceptibility of *K. pneumoniae* isolates was examined and assessed using the disk diffusion method [11]. In this study, antimicrobial disks (Mast Group, Merseyside, UK) including cefepime (FEP, 30 *μ*g), cefpodoxime (CPD, 30 *μ*g), ceftazidime (CAZ, 30 *μ*g), cefotaxime (CTX, 30 *μ*g), piperacillin/tazobactam (PTZ, 100/10 *μ*g), piperacillin (PIP, 100 *μ*g), aztreonam (ATM, 30 *μ*g), gentamicin (GEN, 10 *μ*g), ertapenem (ETP, 10 *μ*g), imipenem (IPM, 10 *μ*g), meropenem (MEM, 10 *μ*g), doripenem (DOR, 10 *μ*g), fosfomycin/trometamol (FOT, 200 *μ*g), tigecycline (TGC, 15 *μ*g), amikacin (AK, 30 *μ*g), trimethoprim-sulfamethoxazole (TS, 2.5 *μ*g), ciprofloxacin (CIP, 5 *μ*g), Minocycline (MN, 15 *μ*g) and nalidixic acid were used.

Since CLSI has not published any approved guideline for *fosfomycin* breakpoints in *K. pneumoniae*, FDA fosfomycin susceptibility breakpoints for Enterobacteriaceae were used as disk diffusion interpretation criteria.

The CLSI recommendations 2020 were used to evaluate the MICs of 7 antibiotics, including colistin, meropenem, imipenem, ciprofloxacin, cefepime, ceftazidime, and cefotaxime, using the broth microdilution technique [11]. The quality of the test was confirmed using *Escherichia coli* ATCC 25922 in the role of control.

### 2.4. Detection of Extended-Spectrum *β*-Lactamase (ESBL)

We used the method of the combination disk diffusion (CDDT) to investigate ESBL production. Antimicrobial disks containing ceftazidime (CAZ) and cefotaxime (CTX) alone and combined disks consisting of ceftazidime 30 *μ*g and clavulanic acid 10 *μ*g and cefotaxime 30 *μ*g and clavulanic acid 10 *μ*g were also used. This test was validated by *K. pneumoniae* ATCC700603 and *E. coli* ATCC 25922 in the role of positive and negative standard controls for the production of ESBL, respectively [12].

### 2.5. PCR Detection of the Genes Linked to Pathogenicity

With the usage of the DNA extraction kit (Roche, Germany, Lot. No. 10362400), genomic DNA was extracted from single bacterial colonies, which were cultured on primary culture plates as stated in the manufacturer's instructions. With the use of specific primers used in prior researches, PCR analysis was done to detect virulence genes (*iucB*, *iutA*, *iroN*, *kfu*, *allS*, *fimH*, *ybtS*, *mrkD*, and *entB*) (Table 1), and the results were authenticated by sequencing. A total volume of 25 *μ*l reaction solution was used for PCR which contained 12.5 *μ*l of 2× Master Mix (SinaClon, Iran, CAT. No., PR901638), 1 *μ*l of 10 pmol of each primer, 2 *μ*l (20 ng) of DNA template, and 8.5 *μ*l of sterile distilled water. The amplification reactions were done by thermal cycler (Eppendorf, MasterCycler Gradient, Germany). In the first phase, denaturation at 94°C for 5 min was done. Then, 36 cycles of denaturation at 94°C for 45 s were performed, and in the next stage, annealing at 50-60°C for 45 s was done. Then, extension at 72°C for 45 s and a final extension at 72°C for 5 min were carried out [13].

The electrophorese of PCR products was done by 1–1.5% agarose gel; then, they were visualized by the use of DNA SafeStain (SinaClon, Tehran, Iran), and in the next stage, they were photographed under UV light. Positive control isolates were generously provided.

### 2.6. PCR Detection of Carbapenemases and Genes Related to Extended-Spectrum *β*-Lactamases

PCR assay with specific primers (used in prior studies) was done to investigate genes linked to ESBL-encoding (*bla*_SHV_, *bla*_TEM_, *bla*_PER_, *bla*_CTX-M_, and *bla*_VEB_), class D (*bla*_OXA-48_), class B (*bla*_VIM_, *bla*_NDM_, and *bla*_IMP_), and class A (*bla*_KPC_ and *bla*_GES_) carbapenemase gene primers (Table 1). Subsequently, PCR results were validated by sequencing. The Medical Microbiology Department of SBMU generously donated positive control isolates.

### 2.7. Statistical Analysis

The findings were interpreted using the Statistical Package for Social Sciences (SPSS) software version 19 for Windows. Qualitative data is expressed using numbers and percentages. Fisher's exact test and Pearson's chi-square test are used to compare categorical data.

## 3. Results

### 3.1. Patients and Bacterial Isolates

Totally, 52 *K. pneumoniae* isolates, which were nonduplicated, were obtained from 21 females (40.4%) and 31 men (59.6%). The patients' ages varied from one to eighty-six years of age. The specimen sources of isolates included tracheal aspirates (3.8%), wound (5.8%), urine (7.7%), blood (13.5%), and sputum (69.2%, 36/52).

### 3.2. Antimicrobial Susceptibility

The results of the antibiotic susceptibility test of all *K. pneumoniae* isolates (*n* = 52) are summarized in Table 2. The results of antimicrobial susceptibility illustrated that the lowest levels of resistance were against Minocycline (13.5%), fosfomycin (21.2%), and colistin (28.8%). Besides, 44 (84.6%), 7 (13.5%), and 1 (1.9%) isolates were MDR, XDR, and PDR, respectively.

### 3.3. ESBL Phenotype

The CDDT phenotypic test revealed that 44 isolates (84.6%) were ESBL-producing.

### 3.4. PCR Results of Virulence-Associated Genes

**Figure 1:**
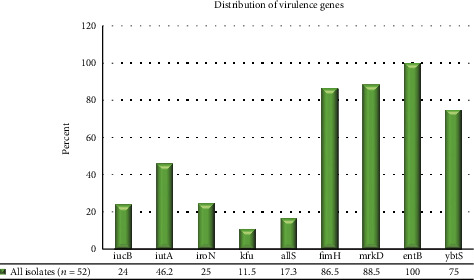
The distribution of virulence genes among all *K. pneumoniae* isolates.

The PCR analysis of all isolates depicted that *iucB*, *iutA*, *iroN*, *kfu*, *allS*, *fimH*, *ybtS*, *mrkD*, and *entB* were carried by 12 (24%), 24 (46.2%), 13 (25%), 6 (11.5%), 9 (17.3%), 45 (86.5%), 39 (75%), 46 (88.5%), and 52 (100%) isolates, respectively (Figure 1).

### 3.5. PCR Results of Antimicrobial Resistance Genes

The PCR analysis of all isolates illustrated that 44 isolates (84.6%) carried ESBL-encoding determinants and 37 (71.2%), 28 (53.8%), and 21 (40.4%) of isolates were positive for genes linked to *bla*_TEM_, *bla*_SHV_, and *bla*_CTX-M_. The *bla*_OXA-48_ gene was carried by 5 isolates (9.6%), although none of the isolates were positive for the existence of *bla*_KPC_, *bla*_IMP_, and *bla*_VIM_. Additionally, 6 isolates (11.5%) were positive *bla*_NDM_ gene.

Besides, the resistance to imipenem was observed among all of the NDM-1 producing *K. pneumoniae* (*p* ≤ 0.05).

## 4. Discussion

*K. pneumoniae* results in a broad spectrum of infections by colonizing and spreading in the human body with the utilization of quite a few virulence factors.

In this study, the patients' ages varied from one to eighty-six years of age. However, there was no noteworthy relationship among the age of the patients and getting infected with *K. pneumoniae*.

The scientific community is concerned about this bacteria's rising antibiotic resistance in recent years.

Above all, the rise in resistance to quite a few antibiotics among *K. pneumoniae* isolates is posing a huge concern in the world.

Heidary et al. (Iran) [14] in a meta-analysis and systematic review article reported that the isolates which were drug-resistant *K. pneumoniae* are highly prevalent in Iran. Due to that report, resistance to ampicillin (82.2%) and aztreonam (55.4%) was indicated to be the most prevalent resistance among *K. pneumoniae* isolates [14]. On the contrary, in the current study, 63.5% of isolates were resistant against aztreonam, and the greatest proportion of resistance among the *K. pneumoniae* isolates was found against piperacillin (96.2%), cefotaxime (92.3%), ceftazidime (90.4%), nalidixic acid (88.5%), and ciprofloxacin (82.7%).

Also, in a research performed by Hashemi et al. (Iran), the greatest rate of resistance among the *K. pneumoniae* isolates was found against ceftazidime (60.2%), cefotaxime (60.2%), piperacillin (60.2%), and ciprofloxacin (55.5%) [9].

In this study, antimicrobial susceptibility tests revealed that the resistance against imipenem was prevalent in 65.4% of isolates (Table 2) which is lesser than the result of a study performed by Shahcheraghi et al. (100%) in 2018, Iran [15], and research done by Remya et al. (87.8%) in 2019, India [16]. However, the prevalence of hvKP stated by Moghadampour et al. (57.5%) in 2018, Iran, and Hashemi et al. (24%) in 2014, Iran [17], was lower than those of our study. Therefore, the percentage of resistance against imipenem has fluctuated from 2018.

In this research, antimicrobial susceptibility tests revealed that 28.8% of isolates were resistant to colistin (Table 2) which is lower rather than the result of a study performed by Rad et al. (31.7%) in 2020, Iran [18], and is more than the result of a research performed by Bir et al. (14.6%) in 2022, India [19], and a study done by Ballen et al. (2%) in 2021, Spain [20]. Thus, resistance to colistin has relatively decreased between 2018 and 2022.

Additionally, another global issue is the prevalence of ESBL-producing isolates of *K. pneumoniae* that its rate differs among different countries. Besides, the reports show that the prevalence of ESBL-producing *K. pneumoniae* is increasing over time in Iran [21]. In this research, 84.6% of isolates were ESBL-producing *K. pneumoniae* which was almost similar to results of a study performed by Moghadampour et al. (88.2%) in 2018, Iran [17], but the prevalence of ESBL-producing isolates in our research was significantly higher than those reported by Ballen et al. (43.3%) in 2021, Spain [20]; El-Domany et al. (40%) in 2020, Egypt [8]; Remya et al. (59.9%) in 2019, India [16]; Khaertynov et al. (50%) in 2018, Russia [22]; Kus et al. (71.7%) in 2017, Turkey [23]; Hashemi et al. (62.3%) in 2014, Iran [9]; Feizabadi et al. (69.7%) in 2009, Iran [24]; and Shahcheraghi et al. (34.5%) in 2007, Iran [15].

In the current study, among all *K. pneumoniae* isolates investigated (*n* = 52), 1 (1.9%), 7 (13.5%), and 44 (84.6%) isolates were PDR, XDR, and MDR, respectively. Generally, according to other studies, a considerable increase in the percentage of multidrug resistance can be seen from 2019, but the amount of extensive drug resistance and pandrug resistance has shown some variations which can be due to the differences in geographical locations, sanitation levels, and antibiotic prescribing trends in hospitals. By way of illustration, in a research performed by Ballen et al. (Spain) [20], 40.2% of *K. pneumoniae* were MDR and only 1.6% of them were XDR. In a study done by Shadkam et al. (Iran) [25], 67% of *K. pneumoniae* were MDR and 11% of them were XDR, but no PDR isolate was reported. In a research performed by Ahmed et al. (Bangladesh) [26], 39.1% of *K. pneumoniae* were MDR, 21.7% of them were XDR, and 3.75% were PDR. In a research performed by El-Domany et al. (Egypt) [8], 42.5% of *K. pneumoniae* were MDR, 35% of them were XDR, and 5% were PDR.

Poirel et al. (Turkey) discovered OXA-48 in *K. pneumoniae* isolates, for the first time [27]. Subsequently, Potron et al. reported the spread of OXA-48 generating *K. pneumoniae* across the Mediterranean area and European countries in 2011 [28]. In 2014, Azimi et al. published the initial report of OXA-48 generating *K. pneumoniae* isolates in Iran [29].

Moreover, a study was done by Sleiman et al. (Lebanon) [30], and they reported the highest number of AMR genes (47 genes) in a *K. pneumoniae* isolate which bring about resistance to all antimicrobial agents which are used widely; hence, this has caused a huge health concern.

Initially, NDM-1 was detected and found in New Delhi, India, by Yong et al. in 2009 [31], and then, numerous case reports were reported in Pakistan and the United Kingdom.

In the current study, 9.6% and 6% of isolates were positive for *bla*_OXA-48_ and *bla*_NDM_ genes, respectively. In a research done by Hashemi et al. (Iran), the prevalence of *bla*_OXA-48_ gene among *K. pneumoniae* isolates was reported 2.4% while the prevalence of *bla*_OXA-48_ among *K. pneumoniae* isolates was reported 58% [9] in a study performed by Candan et al. in 2015, Turkey [32], and 20% in a study performed by Ballen et al. in 2021, Spain [20]. These differences are due to the high prevalence of *bla*_OXA-48_ in Turkey and European countries. Also, the prevalence of *bla*_NDM_ among *K. pneumoniae* isolates reported 5% in a study performed by Ahmed et al. in 2020, Bangladesh [26]; 10% in a study performed by Moghadampour et al. in 2018, Iran; and 23.3% in a study performed by Rad et al. in 2020, Iran [18].

Besides, we evaluated all *K. pneumoniae* isolates for detecting the most important virulence genes, including *iucB*, *iutA*, *iroN*, *kfu*, *allS*, *fimH*, *ybtS*, *mrkD*, and *entB* genes.

Prior studies demonstrate that *entB* gene which is responsible for enterobactin metabolism is highly prevalent in *K. pneumonia* isolates as it is one of the most essential virulence factors among Enterobacteriaceae. The prevalence of *entB* was above 85% in a research performed by Fatima et al. (100%, Balochistan) [33]; Ballen et al. (100%, Spain) [20]; Kus et al. (96.2%, Turkey) [23]; Remya et al. (95%, India) [16]; and Ali and Al-kakei (85.7%, Iraq) [34]. In our research, all of the isolates were positive for *entB* gene.

Earlier reports demonstrate that *YbtS* is the second common siderophore in *K. pneumoniae* after *entB* [7]. In the present study, this gene was observed in 75% of isolates. In China and Algeria, the presence of *YbtS* gene was 83.7% and 46.3%, respectively [35]. Also, the prevalence of *ybts* gene was reported 82.85% by Ali and Al-kakei (Iraq) [34].

Most *K. pneumoniae* isolates express fimbrial adhesins (FimH and MrkD) [36]. In this study, *fimH* gene (type 1 fimbriae) was presented in 86.5% of isolates. The prevalence of *fimH* was above 80% in a research performed by Fatima et al. (100%, Balochistan) [33], Ballen et al. (98.4%, Spain) [20], Zhang et al. (93%, Taiwan) [37], Remya et al. (89.1%, India) [16], and Zhang et al. (85.5%, China) [38].

Also, *mrkD* gene (type 3 adhesin) was presented in 88.5% of isolates. The prevalence of *mrkD* was above 80% in a research performed by Ballen et al. (98.4%, Spain) [20], Kus et al. (83%, Turkey) [23], and Ali and Al-kakei (82.9%, Iraq) [34].

In the current study, 24% of isolates carried *iucB* gene which was reported 23.3%, 8%, and 5.4% by Zhang et al. (Taiwan) [37], Amina and Raheem (Iraq) [39], and Remya et al. (India) [16], respectively.

Furthermore, 25% of isolates carried *iroN* gene which was reported 25% by Khaertynov et al. (Russia) [22].

Also, 46.2% of isolates carried *iutA* gene which was reported 68.6%, 12%, and 5% by Ali and Al-kakei (Iraq) [34], Candan et al. (Turkey) [32], and Kus et al. (Turkey) [23], respectively.

Additionally, *kfu* gene is responsible for coding the iron uptake system and is associated with capsule formation and invasiveness [7, 40, 41]. In the present study, 11.5% of isolates carried this gene which was reported 34.2% and 27.8% by Remya et al. (India) [16] and Ali and Al-kakei (Iraq) [34], respectively.

In this study, the prevalence of allantoin metabolism-associated gene (*allS*) was 17.3% which was reported 14%, 12%, 8.6%, and 6.5% by Zhang et al. (Taiwan) [37], Amina and Raheem (Iraq) [39], Ali and Al-kakei (Iraq) [34], and Zhang et al. (China) [38], respectively, while none of the *K. pneumoniae* isolates carried *allS* in a study performed by Candan et al. (Turkey) [32].

## 5. Conclusions

This study evaluated the detection of virulence factor genes among *K. pneumoniae* isolates. The treatment of these infections is challenging due to the presence of particular virulence factors and the rise of antibiotic resistance. Therefore, the current study accentuates the necessity of finding new and efficient solutions for stopping the increase of antibiotic resistance considering the high prevalence of antibiotic resistance, especially high prevalence of *β*-lactamase-encoding genes, among these isolates with high rate of virulence factors. Above all, new and dependable diagnostic methods for detecting XDR and PDR *K. pneumoniae* isolates should be established.

## Figures and Tables

**Table 1:** PCR primers used in this study.

Target virulence genes	Primer sequence (5′-3′)	Reference
*iucB*	F: ATGTCTAAGGCAAACATCGT	[42]
	R: TTACAGACCGACCTCCGTGA	
*iutA*	F: GGGAAAGGCTTCTCTGCCAT	[42]
	R: TTATTCGCCACCACGCTCTT	
*iroN*	F: AAGTCAAAGCAGGGGTTGCCCG	[42]
	R: TGACGCCGACATTAAGACGCAG	
*fimH*	F: TGCTGCTGGGCTGGTCGATG	[43]
	R: GGGAGGGTGACGGTGACATC	
*mrkD*	F: AAGCTATCGCTGTACTTCCGGCA	[43]
	R: GGCGTTGGCGCTCAGATAGG	
*entB*	F: GTCAACTGGGCCTTTGAGCCGTC	[43]
	R: TATGGGCGTAAACGCCGGTGAT	
*ybtS*	F: GACGGAAACAGCACGGTAAA	[43]
	R: GAGCATAATAAGGCGAAAGA	
*allS*	F: CCGAAACATTACGCACCTTT	[43]
	R: ATCACGAAGAGCCAGGTCAC	
*kfu*	F: GGCCTTTGTCCAGAGCTACG	[43]
	R: GGGTCTGGCGCAGAGTATGC	
*bla*_TEM_	F: TCGGGGAAATGTGCGCG	[44]
	R: TGCTTAATCAGTGAGGCACC	
*bla*_SHV_	F: TTAGCGTTGCCAGTGCTC	[45]
	R: GGTTATGCGTTATATTCGCC	
*bla*_CTX-M_	F: CGCTTTGCGATGTGCAG	[24]
	R: ACCGCGATATCGTTGGT	
*bla*_PER_	F: CCTGACGATCTGGAACCTTT	[46]
	R: GCAACCTGCGCAATGATAGC	
*bla*_VEB_	F: CGACTTCCATTTCCCGATGC	[47]
	R: GGACTCTGCAACAAATACGC	
*bla*_GES_	F: TTGCAATGTGCTCAACGTTC	[48]
	R: TAGTTGTATCTCTGAGGTCG	
*bla*_KPC_	F: CGTCTAGTTCTGTCTTG	[49]
	R: CTTGTCATCCTTGTTAGGCG	
*bla*_VIM_	F: GATGGTGTTTGGTCGCATA	[12]
	R: CGAAATGCGCAGCACCAG	
*bla*_IMP_	F: GGAATAGAGTGGCTTAATTCTC	[48]
	R: CCAAACYACTAAGTTATCT	
*bla*_SPM_	F: GCCGTTTGAAAATCTGGGTAC	[50]
	R: CCTTCGCTTCAGATCCTCG	
*bla*_NDM_	F: GGTTTGGCGATCTGGTTTTC	[52]
	R: CGGAATGGCTCATCACGATC	
*bla*_OXA-48_	F: AAGGAATGGCAAGAAAACAAAA	[51]
	R: CCATAATCGAAAGCATGTAGCA	

**Table 2:** Antimicrobial susceptibility test results for *K. pneumoniae* isolates.

Antibiotics (*n* = 52)	Sensitive no. (%)	Intermediate no. (%)	Resistant no. (%)
Aztreonam (ATM, 10 *μ*g)	7 (13.5%)	12 (23.1%)	33 (63.5%)
Gentamicin (GM, 10 *μ*g)	12 (23.1%)	8 (15.4%)	32 (62.5%)
Ciprofloxacin (CIP, 30 *μ*g)	5 (9.6%)	4 (7.7%)	43 (82.7%)
Fosfomycin (FOS, 200 *μ*g)	39 (75%)	2 (3.8%)	11 (21.2%)
Trimethoprim sulfamethoxazole (TS, 30 *μ*g)	14 (26.9%)	0	38 (73.1%)
Amikacin (AK, 30 *μ*g)	14 (26.9%)	4 (7.7%)	34 (65.4%)
Cefotaxime (CTX, 30 *μ*g)	0	4 (7.7%)	48 (92.3%)
Ceftazidime (30 *μ*g)	3 (5.8%)	2 (3.8%)	47 (90.4%)
Cefepime (FEP, 30 *μ*g)	3 (5.8%)	15 (28.8%)	34 (65.4%)
Nalidixic acid (NA, 30 *μ*g)	0	6 (11.5%)	46 (88.5%)
Piperacillin (PIP, 100 *μ*g)	0	2 (3.8%)	50 (96.2%)
Minocycline (MN, 15 *μ*g)	36 (69.2%)	9 (17.3%)	7 (13.5%)
Doripenem (DOR, 10 *μ*g)	9 (17.3%)	7 (13.5%)	36 (69.2%)
Ertapenem (ETP, 10 *μ*g)	13 (25%)	3 (5.8%)	36 (69.2%)
Imipenem (IMI, 10 *μ*g)	15 (28.8%)	3 (5.8%)	34 (65.4%)
Meropenem (MEM, 10 *μ*g)	22 (42.3%)	0	30 (57.7%)
Colistin (COL)	37 (71.2%)	0	15 (28.8%)
Piperacillin/tazobactam (PTZ, 100/10 *μ*g)	11 (21.2%)	7 (13.5%)	34 (65.4%)

## Data Availability

All the data generated or analyzed during this study were included in this article.
